# Exploring The Migration Profiles of Primary Healthcare Users in South Africa

**DOI:** 10.1007/s10903-016-0535-7

**Published:** 2016-12-01

**Authors:** Jo Vearey, Thea de Gruchy, Mphatso Kamndaya, Helen L. Walls, Candice M. Chetty-Makkan, Johanna Hanefeld

**Affiliations:** 1 African Centre for Migration & Society, University of the Witwatersand, PO Box 76, Wits, Johannesburg, 2050 South Africa; 2 London School of Hygiene & Tropical Medicine, London, UK; 3 The Aurum Institute, Johannesburg, South Africa

**Keywords:** Migration, Public healthcare, Access to healthcare, South Africa

## Abstract

South Africa’s public healthcare system responses seldom engage with migration. Our exploratory study investigates migration profiles and experiences of primary healthcare (PHC) users. A cross-sectional survey involving non-probability sampling was conducted with 229 PHC users at six purposively selected PHC clinics in three districts of SA. The survey captured socio-demographic information, migration histories, and PHC experiences. Chi square and Fischer’s exact tests were used to compare categorical variables, whilst Mann–Whitney U tests compared continuous variables between groups. Most PHC users were migrants (22% internal South African; 45% cross-border) who generally move for reasons other than healthcare seeking. Length of time accessing services at a specific clinic was shown to be key in describing experiences of PHC use. Understanding population movement is central to PHC strengthening in SA and requires improved understanding of mobility dynamics in regard to not just nationality, but also internal mobility and length of stay.

## Background

South Africa is home to diverse historical and contemporary population movements, predominantly associated with livelihood seeking [1, 2]. In line with global averages, and contrary to popular opinion, latest estimates suggest that between 3 and 4% of South Africa’s population are non-nationals, mostly from other sub-Saharan African countries [3, 4], and twice as many people—approximately 7% of the population (3.7 million)—are internal migrants, individuals who have moved between South Africa’s nine provinces [3]. Internal and cross-border migrants are unevenly distributed across the country: whilst the majority of cross-border migrants are located in Johannesburg, Cape Town, Durban and the Vhembe District (a district in Limpopo province that borders Zimbabwe), where internal migrants are mostly found in large and small urban areas [3, 4]. Despite this data, and existing evidence that clearly highlights the prevalence of diverse population movements within South Africa, the provision of public healthcare services—upon which the majority of the population relies—rarely engages with mobility or migration in any form for example, [5, 6].

Recent research has explored the ways in which different forms of patient travel, particularly ‘high-end’ medical tourism—which is when individuals move across borders to access private healthcare–impact healthcare systems [7–12]. This research highlights gaps in knowledge about medical travel within and between lower and middle-income country contexts, such as South Africa [13]. In response to this, a mixed-methods study was undertaken in 2015 to explore methodologies for assessing the impact of medical travel on the South African public healthcare system see [6]. The analysis presented in this paper constitutes one aspect of the broader study, drawing on an exploratory cross-sectional survey that utilised a non-traditional sampling approach in order to investigate the migration profiles of primary healthcare (PHC) users and the ways in which this mediates experiences of PHC services. PHC is the first level of the South African public healthcare system, providing a range of free services to all, such as antenatal care, immunisations, and chronic treatment—including HIV and TB [14].

## Methods

### Study Setting

This paper draws on an exploratory survey conducted with a non-probability sample of 127 South African nationals and 102 non-nationals generated across six PHC clinics (range of 30–46 participants at each clinic, see Table 1), located in three districts of South Africa. The locations and names of these clinics are blinded in the reporting of results, and have been aggregated by location type in the reporting of results, as follows: urban (U); peri-urban (PU); rural (R); and, cross-border (CB).

**Table 1:** Description of socio-demographics and use of public healthcare by nationals and non nationals in six clinics in South Africa (N = 229)

Variables	RSA (n = 127)	Non-RSA (n = 102)	p value
	n (%) or median IQR, n	n (%) or median IQR, n	
Age in years	34 (25–41), 127	29 (26–34), 99	0.01^a^
Gender^e^			0.53^b^
Female	106 (84.1)	86 (84.3)	
Male	20 (15.9)	15 (14.7)	
Clinic			<0.001^c^
U1	13 (10.2)	20 (19.6)	
U2	11 (8.7)	33 (32.4)	
PU1	32 (25.2)	4 (3.9)	
PU2	14 (11.0)	16 (15.7)	
CB	21 (16.5)	25 (24.5)	
R	36 (28.4)	4 (3.9)	
Documentation			<0.001^c^
Undocumented	0 (0.0)	38 (37.2)	
Citizen with ID	125 (98.4)	1 (1.0)	
Citizen without ID	2 (1.6)	0 (0.0)	
Passport not RSA	0 (0.0)	6 (5.9)	
Asylum seeker	0 (0.0)	25 (24.5)	
Refugee	0 (0.0)	1 (1.0)	
TRP: visitor	0 (0.0)	5 (4.9)	
TRP: student	0 (0.0)	1 (1.0)	
TRP: work	0 (0.0)	21 (20.5)	
Permanent residence	0 (0.0)	2 (2.0)	
Unknown	0 (0.0)	1 (1.0)	
Other	0 (0.0)	1 (1.0)	
Why this clinic^d^			0.34^c^
Proximity	71 (71.0)	59 (73.8)	
Don’t ask for ID	0 (0.0)	2 (2.4)	
Staff are nice	20 (20.0)	11 (13.8)	
Other	9 (9.0)	8 (10.0)	
Length of time at this clinic			<0.001^c^
First visit	8 (6.3)	9 (8.8)	
<6 months	15 (11.8)	22 (21.6)	
7 < 12 months	7 (5.5)	20 (19.6)	
12 < 24 months	14 (11.0)	15 (14.7)	
24 < 36 months	12 (9.5)	6 (5.9)	
>3 years	64 (50.4)	30 (29.4)	
Whole life	7 (5.5)	0 (0.0)	
Problems experienced at this clinic (yes)^f^	52 (41.6)	46 (46.5)	0.47^b^

*TRP* temporary residence permit

^a^Mann–Whitney U test

^b^Chi square test

^c^Fisher’s exact

^d^This analysis excludes n = 49 participants who were not asked this question

^e^Two participants (one South African and one Non-South African) whose gender is missing were excluded from this analysis

^f^Five participants (two South Africans and three Non-South Africans) with missing information were excluded from this analysis

### Sampling

Based on interviews with key informants, two clinics were purposively selected to represent areas of both high and low levels of cross-border migration within each of the three districts. The six clinics reflected urban, peri-urban, rural and cross-border settings. In the absence of a sampling frame, we used a convenience (non-probability) approach to invite PHC users to participate in the survey. Healthcare users waiting in the queue at each clinic were invited at random to participate in a short, administered survey. The participant’s place was saved in the queue and the survey administered in their language of choice—fieldworkers were able to translate the survey from English into Shona, Ndebele, Zulu, Venda, Shangani, French or Lingala—in a private space, most frequently an empty consultation room.

### Measures

The survey was designed to capture basic socio-demographic information, including documentation status. This category refers to whether an individual reported holding a South African Identity Booklet, a passport (South African or other nationality), and/or a valid permit (temporary and permanent residence, asylum seeker, refugee). Individuals with passports but without a valid permit were categorised as ‘undocumented’. Individual migration histories—including length of time in South Africa and current province, and length of time resident at current address—were documented. In addition, engagement with the public healthcare system in South Africa was recorded—including length of time using the PHC clinic at which interviewed, reason for choosing the clinic at which interviewed, and any challenges encountered. The survey tool was based on previous surveys conducted by the African Centre for Migration & Society (ACMS) at the University of the Witwatersrand, including a national longitudinal survey with migrants who made use of public healthcare facilities [15], a cross-sectional survey undertaken with healthcare users at antiretroviral (ART) clinics in Johannesburg [16], and a range of household surveys [17–22]. The primary outcomes that were measured include migration type and length of stay in current place of residence.

### Analysis

All statistical analyses were performed in the statistical software package STATA (version 12, StataCorp LP, College Station, Texas). We performed descriptive statistics; Chi square and Fischer’s exact tests to compare categorical variables, and Mann–Whitney U tests to compare continuous variables between groups. A description of the use of PHC by nationals and non-nationals in the sample is provided, followed by comparisons between migrant type and migration status; these are described below. The study sample consisted of 229 individuals. For some analyses, cases were removed due to missing data; this is noted in the appropriate table.

For Table 1, the sample was divided into two groups defined as South Africans (South African identification) and non-South Africans (no South African Identification) to allow for comparison between the experiences of accessing PHC between South African nationals and non-nationals.

During analysis, two new categories were created. The first – *migrant type –* allowed us to explore whether different migration types were associated with different socio-demographic characteristics or experiences in accessing public healthcare. This classification was included in Table 2 where the sample was divided into three groups—non-migrants, internal migrants and cross border migrants. For this, South Africans were re-classified as either *non-migrant* (those living in their province of birth, n = 75) or *internal migrant* (those living in a different province to that of their birth, n = 52). Cross border migrants remained as those who were non-South African nationals. It is important to note that this crude classification does not allow mobility within a province to be distinguished from cross-border population mobility.

**Table 2:** Pairwise comparison of socio-demographics and use of public healthcare by migrant type in six clinics in South Africa (N = 229)

Variables	Non-migrant (n = 75); A	Internal migrant (n = 52); B	Cross-border migrant (n = 102); C	p value^a^ A vs B	p value^a^ A vs C	p value^a^ B vs C
	n (%) or median IQR, n	n (%) or median IQR, n	n (%) or median IQR, n			
Age in years	35 (25–44), 75	31.5 (25–37), 52	29 (26–34), 99	0.23^a^	0.004^a^	0.23^a^
Gender^e^				0.05	0.50^b^	0.37^b^
Female	67 (89.3)	39 (76.5)	86 (85.1)			
Male	8 (10.7)	12 (23.5)	15 (14.9)			
Clinic				<0.001^c^	<0.001^c^	<0.001^c^
U1	5 (6.7)	8 (15.4)	20 (19.6)			
U2	2 (2.7)	9 (17.3)	33 (32.4)			
PU1	11 (14.7)	21 (40.4)	4 (3.9)			
PU2	7 (9.3)	7 (13.5)	16 (15.7)			
CB	19 (25.3)	2 (3.9)	25 (24.5)			
R	31 (41.3)	5 (9.6)	4 (3.9)			
Documentation				0.24^c^	<0.001^c^	<0.001^c^
Undocumented	0 (0.0)	0 (0.0)	38 (37.2)			
Citizen with ID	73 (97.3)	52 (100.0)	1 (1.0)			
Citizen without ID	2 (2.7)	0 (0.0)	0 (0.0)			
Passport not RSA	0 (0.0)	0 (0.0)	6 (5.9)			
Asylum seeker	0 (0.0)	0 (0.0)	25 (24.5)			
Refugee	0 (0.0)	0 (0.0)	1 (1.0)			
TRP: visitor	0 (0.0)	0 (0.0)	5 (4.9)			
TRP: student	0 (0.0)	0 (0.0)	1 (1.0)			
TRP: work	0 (0.0)	0 (0.0)	21 (20.5)			
Permanent residence	0 (0.0)	0 (0.0)	2 (2.0)			
Unknown	0 (0.0)	0 (0.0)	1 (1.0)			
Other	0 (0.0)	0 (0.0)	1 (1.0)			
Why this clinic^d^				0.76^c^	0.43^c^	0.93^c^
Proximity	39 (68.4)	32 (74.4)	59 (73.8)			
Don’t ask for ID	0 (0.0)	0 (0.0)	2 (2.5)			
Staff are nice	13 (22.8)	7 (16.3)	11 (13.8)			
Other	5 (8.8)	4 (9.3)	8 (10.0)			
Length of time at this clinic				0.05^c^	<0.001^c^	0.05^c^
First visit	2 (2.7)	6 (11.5)	9 (8.8)			
<6 months	7 (9.3)	8 (15.4)	22 (21.6)			
7 < 12 months	4 (5.3)	3 (5.8)	20 (19.6)			
12 < 24 months	8 (10.7)	6 (11.5)	15 (14.7)			
24 < 36 months	4 (5.3)	8 (15.4)	6 (5.9)			
>3 years	44 (58.7)	20 (38.5)	30 (29.4)			
Whole life	6 (8.0)	1 (1.2)	0 (0.0)			
Problems experienced at this clinic (yes)^f^	27 (36.5)	25 (49.0)	46 (46.5)	0.16^b^	0.19^b^	0.77^b^

^a^Mann–Whitney U test

^b^Chi square test

^c^Fisher’s exact

^d^This analysis excludes n = 49 participants who were not asked this question

^e^Two participants (one South African and one Non-South African) whose gender is missing were excluded from this analysis

^f^Five participants (two South Africans and three Non-South Africans) with missing information were excluded from this analysis

A second category (Table 3) representing *length of stay* which was defined as the length of time resident in the place of interview [19, 21]. For Table 3, the sample was divided into three groups that included new arrivals (resident for less than 1 year within the same location of the clinic where the survey took place), migrants (being resident for 1–5 years within the same location of the clinic where the survey took place), or long-term residents (being resident for more than 5 years within the same location of the clinic where the survey took place). These new categories were used to explore whether different lengths of stay were associated with different socio-demographic characteristics or experiences in accessing public healthcare.

**Table 3:** Pairwise comparison of socio-demographics and use of public healthcare by different users based on length of time resident at current address in six clinics in South Africa (N = 221)

Variables	Recent arrivals <1 year (n = 58); A	Migrants 1–5 years (n = 59); B	Long-term residents >5 years (n = 104); C	p value^a^ A vs B	p value^a^ A vs C	p value^a^ B vs C
	n (%) or median IQR, n	n (%) or median IQR, n	n (%) or median IQR, n			
Age in years	27.5 (23–37), 58	28 (25–34), 59	34 (29–40), 104	0.53^a^	0.001^a^	< 0.001^a ^
Gender^e^				0.59^b^	0.31^b^	0.50^b^
Female	52 (89.7)	51 (86.4)	85 (83.3)			
Male	6 (10.3)	8 (13.6)	17 (16.7)			
Clinic				< 0.001^c^	< 0.001^c^	0.15^c^
U1	4 (6.9)	10 (17.0)	19 (18.3)			
U2	7 (12.1)	18 (30.5)	19 (18.3)			
PU1	7 (12.1)	5 (8.5)	23 (22.1)			
PU2	2 (3.5)	10 (17.0)	12 (11.5)			
CB	13 (22.4)	12 (20.3)	20 (19.3)			
R	25 (43 1)	4 (6.8)	11 (10.6)			
Documentation				0.01	0.03^c^	< 0.001^c^
Undocumented	11 (19.0)	17 (28.8)	8 (7.7)			
Citizen with ID	38 (65.5)	19 (32.2)	64 (61.5)			
Citizen without ID	0 (0.0)	0 (0.0)	2 (1.9)			
Passport not RSA	1 (1.7)	2 (3.4)	3 (2.8)			
Asylum seeker	4 (6.9)	12 (20.3)	8 (7.7)			
Refugee	0 (0.0)	1 (1.7)	0 (0.0)			
TRP: visitor	2 (3.5)	2 (3.4)	1 (1.0)			
TRP: student	1 (1.7)	0 (0.0)	0 (0.0)			
TRP: work	1 (1.7)	5 (8.5)	15 (14.4)			
Permanent residence	0 (0.0)	1 (1.7)	1 (1.0)			
Unknown	0 (0.0)	0 (0.0)	1 (1.0)			
Other	0 (0.0)	0 (0.0)	1 (1.0)			
Why this clinic^d^				0.55^c^	0.25^c^	0.54^c^
Proximity	34 (66.7)	32 (78.1)	62 (75.6)			
Don’t ask for ID	2 (3.9)	0 (0.0)	0 (0.0)			
Staff are nice	10 (19.6)	7 (17.1)	11 (13.4)			
Other	5 (9.8)	2 (4.9)	9 (11.0)			
Length of time at this clinic				0.01^c^	0.004^c^	<0.001^c ^
First visit	11 (19.0)	3 (5.1)	3 (2.9)			
<6 months	11 (19.0)	13 (22.3)	10 (9.6)			
7 < 12 months	7 (12.1)	9 (15.3)	11 (10.6)			
12 < 24 months	5 (8.6)	14 (23.7)	9 (8.7)			
24 < 36 months	3 (5.2)	10 (17.0)	5 (4.8)			
>3 years	20 (34.5)	10 (17.0)	61 (58.7)			
Whole life	1 (1.7)	0 (0.0)	5 (4.8)			
Problems experienced at this clinic (yes)^f^	18 (33.3)	25 (42.4)	52 (50.5)	0.25	0.04^b ^	0.32^b^

Eight participants had missing information on length of stay

^a^Whitney U test

^b^Chi square test

^c^Fisher’s exact

^d^This analysis excludes n = 49 participants who were not asked this question

^e^Two participants (one South African and one Non-South African) whose gender is missing were excluded from this analysis

^f^Five participants (two South Africans and three Non-South Africans) with missing information were excluded from this analysis

### Ethical Considerations

The research was approved by the University of the Witwatersrand Research Ethics Committee (non-medical), the London School of Hygiene and Tropical Medicine Research Ethics Committee, and the relevant Provincial and District Health Departments.

## Results

### Comparison of Nationals and Non-Nationals Use of PHC

The sample for this analysis included 127 South African nationals and 102 non-nationals. Non-nationals were younger than South African nationals (median age 34 vs 29; p = 0.01). There were no differences in gender distribution between the two groups (p = 0.53), and 192 (84%) of the respondents were female. Regarding reasons for choosing to access a particular clinic, both South African nationals and non-nationals indicated proximity as the dominant reason. There was a difference in documentation status between the two groups (p < 0.001). Most nationals- 125 out of 127 (98.4%)- were documented citizens holding South African Identity Books. Amongst non-nationals, 38 (37.2%) were undocumented, 25 (24.5%) held asylum seeker permits, and 21 (20.5%) held temporary resident permits. There was also a difference between the two groups in terms of the length of time that respondents had been attending the same clinic at which they were interviewed (p < 0.001). More South African nationals compared to non-nationals attended the clinic for at least 2 years (9.5 vs 5.9%), more than 3 years (50.4 vs 29.4%), and whole life (5.5 vs 0.0%) respectively. While, more non-nationals compared to South African nationals attended the same clinic for less than 2 years; clinic for the first time (8.8 vs 6.3%), 0–6 months (21.6 vs 11.8%), 7–11 months (19.6 vs 5.5%) and 12–23 months (14.7 vs 11.0%) respectively. There was no difference between the groups in the reporting of problems experienced at the clinic at which they were interviewed (p = 0.47) (see Table 1).

### Comparison of Non-Migrant, Internal Migrant, Cross-Border Migrants Use of PHC

The analysis for this comparison included 75 non-migrants, 52 internal migrants, and 102 cross-border migrants. Non-migrants were older than cross-border migrants (median age 35 vs 29 years; p = 0.004); there was no association in age between non-migrants and internal migrants. There was a difference in the type of clinic accessed by the three groups. More non-migrants (41.3%) accessed services at the rural clinic when compared to internal migrants (9.6%; p < 0.001) or cross-border migrants (3.9%; p < 0.001). A higher proportion of internal migrants compared to cross-border migrants were at peri-urban clinics (53.9 vs 19.6%; p < 0.001). Non-migrants and internal migrants were South African nationals and the majority had some form of identification. However, differences in documentation status across the groups were evident. More cross-border migrants when compared to non-migrants (p < 0.001) and internal migrants (p < 0.001) were undocumented (37.2 vs 0.0%), asylum seekers (24.5 vs 0.0%) or TRP workers (20.5 vs 0.0%) respectively.

More cross-border migrants when compared to non-migrants attended the clinic at which they were interviewed for less than 2 years; first visit (8.8 vs 2.7%), less than 6 months (21.6 vs 9.3%), 7–11 months (19.6 vs 5.3%), 12–23 months (14.7 vs 10.7%) respectively. However, more non-migrants had been attending this clinic for more than 3 years (58.7%) and their whole life (8.0%) when compared to 29.4% and 0.0% of cross-border migrants respectively.

There was no difference in length of time at the clinic where the interview took place between non-migrants and internal migrants, or between internal migrants and cross-border migrants. No differences were found when comparing reasons for choosing the clinic at which individuals were interviewed between non-migrants and internal migrants (p = 0.76), non-migrants and cross-border migrants (p = 0.43) or internal migrants and cross border (p = 0.93). All three groups indicated proximity as their main reason for choosing the clinic. There were no differences between the three groups in problems being experienced at the clinic (see Table 2).

### Comparison of New Arrivals, Migrants Long-Term Residents Use of PHC

The analysis for this comparison included 58 new arrivals (length of time resident in the place of interview), 59 migrants (resident 1–5 years) and 104 long-term residents (resident more than 5 years). There was a difference in median age between new arrivals, migrants and long-term residents with long-term residents being older than new arrivals (median age 34 vs 28 years; p = 0.001) and migrants (median age 34 vs 28 years; p < 0.001) respectively.

New arrivals, migrants, and long-term residents were unevenly distributed across different clinics. There was a higher proportion of new arrivals at the rural (43.1%) and cross-border (22.4%) clinics. While the proportion of migrants (resident for 1–5 years) were high at urban clinics (47.5%) with more long-term residents being present at urban (36.6%) and peri-urban (33.6%) clinics. A greater proportion of new arrivals (43.1%) were using the rural clinic when compared to migrants (resident for 1–5 years) (6.8%; p < 0.001) and to long-term residents (10.6%; p < 0.001). Compared to new arrivals, a higher proportion of migrants (resident for 1–5 years) were using the urban clinics (47.5 vs 19%; p < 0.01).

A higher proportion of migrants resident in the area for 1–5 years reported being undocumented compared to new arrivals (28.8 vs 19.0%; p = 0.01) and long-term residents (28.8 vs 7.7%; p < 0.001). A lower proportion of migrants resident in the area for 1–5 years reported being citizens with identity documents (32.3%), compared to new arrivals (65.5%; p = 0.01) and long-term residents (61.5%; p < 0.001). A higher proportion of asylum seekers were resident for 1–5 years (20.3%), compared to new arrivals (6.9%; p = 0.01) and long-term residents (7.7%; p < 0.001). A higher proportion of migrants with temporary resident permits were resident for more than 5 years (15.4%), compared to recent arrivals (6.9%; p = 0.03) and migrants resident for 1–5 years (11.9%; p < 0.001).

Differences were found in terms of the length of stay at their current residence and the length of time respondents had been visiting the clinic at which they were interviewed. A higher proportion of long-term residents reported attending the clinic in question for more than 3 years, compared to recent arrivals (58.7 vs 34.5%; p = 0.004) and migrants resident for 1–5 years (58.7 vs17%; p = 0.01). Migrants resident for 1–5 years were more likely to report attending the clinic for less than 2 years than recent arrivals (23.7 vs 8.6%; p = 0.01) and long-term residents (23.7 vs 8.7%; p = 0.004).

No differences were found in reasons for choosing a particular clinic, with proximity again being the most popular reason across the three groups. A smaller number reported that staff attitudes were important. More long-term residents reported experiencing problems at the clinic of interview - mostly relating to complaints associated with long-waiting times^1^ compared to new arrivals (50.5 vs 33.3%; p = 0.04).

## Discussion

All facilities included in the study were found to serve a diverse range of patients including long-term residents, recent arrivals, internal and cross-border migrants. Women account for the majority of public healthcare users surveyed, illustrating the known gendered dimension of healthcare seeking—and associated gendered burden of healthcare-associated challenges—documented elsewhere [23–25]. Recent arrivals were significantly younger than longer-term residents, in line with existing literature—including in South Africa [21]. The demographics of our sample population—generated through a convenience sampling approach and chosen to reflect areas of relatively high as well as relatively low levels of cross-border migration—are also in line with South African migrant profiles that have been documented elsewhere, although not in healthcare settings [for example, 3]. Documentation status amongst our sample was associated with length of stay. Compared to recent migrants, new arrivals and long-term residents were most likely to be documented. This suggests that, on arrival, non-nationals have documents but that these expire and non-nationals ‘overstay’ the duration of their visa, and it takes several years for individuals to regain a documented status—often through a temporary residence permit.

The majority of public healthcare users we surveyed were born in a province (or country) different to where they were interviewed, indicating the prevalence of a migration history within the study sample. There were significant differences between clinic location and classification of migrant groups: a higher proportion of cross-border migrants were found at urban clinics; internal South African migrants at peri-urban clinics; and, non-migrants (born in the province of interview) at the rural facility. Regardless of migration status or length of stay, the majority of respondents reported using a clinic due to its proximity, followed by a smaller number reporting that their choice of clinic is because ‘staff are nice’. This indicates that, when seeking health care, convenience and- to some extent- staff attitudes are paramount. When comparing nationality or migration status, although not statistically significant, a greater proportion of migrants (non-nationals vs nationals, Table 1; internal and cross-border migrants vs non-migrants, Table 2) reported having experienced problems at the clinic at which they were interviewed. When exploring length of stay, however, long-term residents were significantly more likely than recent arrivals to report problems. This suggests three possibilities: (1) that long-term residents are more confident in expressing their dissatisfaction; (2) that recent arrivals have either not yet accrued sufficient PHC user experience to have experienced problems at the clinic; or, (3) have not yet accrued sufficient PHC user experience to consider reporting their experience as problematic. The latter two suggestions are supported by the data showing that length of stay in current residence is associated with length of time accessing PHC services at the clinic of interview (discussed below).

Many users (recent arrivals, migrants and long-term residents) had been resident at their current address for a longer period of time than they reported accessing healthcare at the particular clinic in which they were surveyed (Table 3). These findings show that these respondents did not start accessing healthcare, at least at that particular clinic, at the same time that they moved into the area of interview, suggesting that they did not move in order to access PHC services.

The survey asked users whether they were accessing other healthcare facilities simultaneously; this was almost exclusively reported not to be the case with the majority of users indicating they only make use of the PHC facility at which they were interviewed (data not shown). Together, these findings suggest that whilst most PHC users surveyed have migration histories, they are not moving in order to access PHC. In the survey, non-national PHC users reported moving for other reasons—mostly to seek employment or to join family (data not shown). This suggests that, in line with existing research [26], the majority of PHC users surveyed have not moved in order to access health services.

Respondents who had been resident at their current address for less than 1 year were more likely to be found at the rural clinic. This group—mostly South African nationals—reported using the rural clinic for a longer period of time than they had been living at their current residence, suggesting high levels of local (intra-provincial) mobility of South African nationals within the rural clinic catchment area.

In our sample, recent arrivals and migrants are younger than long-term residents (no difference in age between new arrivals and migrants). Long-term residents have been accessing PHC services for a longer time (>3 years) at the clinic of interview when compared to new arrivals and migrants (1–5 years). An obvious confounder here is the length of time that the person was living in the area of interview. However, our findings do suggest that the PHC users we surveyed have not moved in order to access healthcare. Our findings support existing research that shows that individuals who move are positively selected: they are younger and therefore likely to be healthier than both the population they leave and the population they join, suggesting the presence of a healthy migrant effect [2, 26–28]. Whilst further research is required, these findings are important in challenging the prevalent assumptions that associate migration with poor health and healthcare-seeking [5].

### Study Limitations

The survey described follows a methodology type that aimed to produce a cross-sectional sample that is sufficiently representative of the diverse migration context of South Africa [15, 16, 20], for example, see [29–33]. We undertook statistical analyses similar to those of previous studies that involved a comparable, non-traditional sampling approach, including studies that were also undertaken in the absence of a probability sampling frame and involved small sample sizes [15, 16, 30]. Importantly—and key to this study—such an approach allows for exploratory cross-sectional research in a situation where it was not feasible to develop a probability sampling frame [15, 16, 30], and thus allows for exploratory analysis in a context of resource and time constraints. Previous studies exploring migration have raised concerns relating to possible bias, particularly that associated with nationality, documentation, gender and vulnerability [15]. Whilst we did not collect data on non-responders, the demographic data presented here in our non-probability sampling approach reflect results from other surveys of migrants and non-migrants in SA in each location (Census, 2011), suggesting we obtained a sufficiently representative sample. For example, our study obtained a range of responses describing documentation types similar to that which would be expected based on previous estimates [26, 29]. Importantly, this included reporting of undocumented status—often assumed to be a reason for non-response. In line with global trends relating to gender bias in healthcare-seeking behaviour, women in our sample accounted for more respondents than men. Vulnerability bias, in this case, would refer to individuals who had previously had bad experiences at PHC facilities and, as a result, would not return, and therefore be excluded from our sample. Whilst we recognise this limitation, our sample also included many respondents who reported significant challenges with accessing PHC but still chose to return.

### Conclusion

The findings support and strengthen existing knowledge on migration profiles in South Africa, and provide new insights both empirically in terms of migration profiles and experiences in PHC settings in SA, and also methodologically in terms of conducting exploratory research in resource-constrained settings. The findings also raise further research questions relating to how the dynamics of migration and mobility affect the experiences of PHC users in South Africa. The results highlight the high prevalence of a migratory status amongst PHC users—regardless of nationality—and emphasise the importance of gaining improved understanding of local (intra-provincial), internal (inter-provincial) as well as external (cross-border) mobility. The findings suggest that the population making up the rural sample is associated with higher levels of local (intra-provincial) mobility than the urban and peri-urban samples; further research is required here.

In line with prior research comparing the experiences of different South African and cross-border migrants [19, 21, 26], our findings suggest that nationality alone does not explain the different experiences of PHC users. Our study indicates that both length of time accessing a particular PHC facility and length of stay in current residence are important in explaining the differences in experience of PHC users. This does not mean that healthcare responses should ignore nationality and the ways in which this mediates the experiences of PHC users—including through language, culture, documentation status and the attitudes of staff. To the contrary: our findings highlight that the diverse migration profiles of all healthcare users must be considered within the strengthening PHC provision in South Africa. In support of this, we suggest that future research and associated health systems strengthening should move away from “methodological nationalism”—a focus solely on nationality when exploring population movements—in the ways that migration and mobility are conceptualised, measured and responded to [19, 34, 35]. Instead, engagement with population movement for strengthening health systems needs to consider the complexities of migration histories, including where these relate to internal migration as well as cross-border migration.

Critically, this paper highlights the need for further research to improve our understanding of the dynamics of population movement amongst users of the South African PHC system, with implications for the design and implementation of improved public health system responses in both South Africa and the southern African region. Key here are concerns relating to the ways in which migration and mobility influence access to MCH services, continuity of access to chronic treatment provided through PHC services, and associated concerns surrounding the successful control of communicable and non-communicable diseases in the region. We hope that the findings from this study will go some way to supporting efforts towards strengthening PHC services in South Africa, including through the current PHC re-engineering and National Health Insurance (NHI) processes.

## Footnotes

1 Data not presented here.
